# Hepatocellular Carcinoma Misdiagnosed as a Liver Abscess – A Story of Misdiagnosis and Long-Term Malignant Disease Control

**DOI:** 10.7759/cureus.12636

**Published:** 2021-01-11

**Authors:** Ahmed Mahmoud, Kellee Slater

**Affiliations:** 1 Surgery, University of Queensland, Brisbane, AUS

**Keywords:** hepatocellular carcinoma (hcc), transarterial chemoembolization (tace), liver masses, liver abcess

## Abstract

Hepatocellular carcinoma (HCC) is the most common liver malignancy. The presentation of HCC is highly variable which can delay diagnosis. However, the early diagnosis of HCC can significantly improve prognosis. A rare presentation of a patient with a new diagnosis of HCC with sepsis is described. A 56-year-old male presented septic with abdominal pain and a background of a chronic foot infection. The septic screen identified echocardiographic evidence of vegetations on the aortic and mitral leaflets. Also, an ultrasound of the abdomen identified multiple hypoechoic lesions suspicious for liver abscesses. Multiple attempts of ultrasound-guided aspiration of liver lesions were unsuccessful and he had a tumultuous course with recurring fevers over a period of six months. The diagnosis of HCC was eventually confirmed after the lesion eroded into his anterior gastric wall which caused an upper gastrointestinal bleed. He responded well to transarterial chemoembolization (TACE) and palliative radiation therapy. At four-year follow-up and after 11 TACE procedures, he is well without liver dysfunction and independent with daily activities.

## Introduction

Hepatocellular carcinoma (HCC) is the most common primary hepatic malignancy [1]. The incidence of HCC is on the rise worldwide due the spread of chronic hepatitis B and C infections [2]. HCC can be locally invasive as well as metastasize to other organs. The initial presentation of a patient with the disease can be variable [3]. Patients can present with symptoms related to growth of tumour such as vague upper abdominal pain, weight loss, or fatigue or may present with symptoms of liver cirrhosis [4]. HCC is often diagnosed at the advanced stages of the disease and has a poor prognosis.

We present the case of a 56-year old male with a histologically confirmed TNM stage T4B HCC which ruptured and eroded into the anterior gastric wall due to misdiagnosis as an abscess. This case illustrates the importance of assessing a patient’s clinical response to treatment and considering alternative diagnoses if the patient’s clinical course is not following the expected path.

## Case presentation

A 56-year-old male presented to a regional hospital with sepsis. He was febrile with abdominal pain and a suspected source of a chronic right foot ulcer. He had a past medical history of type two diabetes mellitus which was well controlled with dietary modification and a haemaglobin A1C (HbA1C) of 5.6. He took no regular medications. He was an ex-smoker of 20 pack-year history and once a fortnight would engage in excessive alcohol intake. He denied any intravenous drug use, tattoos, piercings, high-risk sexual activity, or previous blood transfusions. In his family history it was noted that one of his brothers passed away from HCC at a similar age and his other brother has been recently diagnosed with HCC but alive. On the initial examination, he was febrile, tachycardiac, and demonstrated signs of cardiac failure which were fluid overload and shortness of breath. He also had a complex heart murmur.

**Figure 1:**
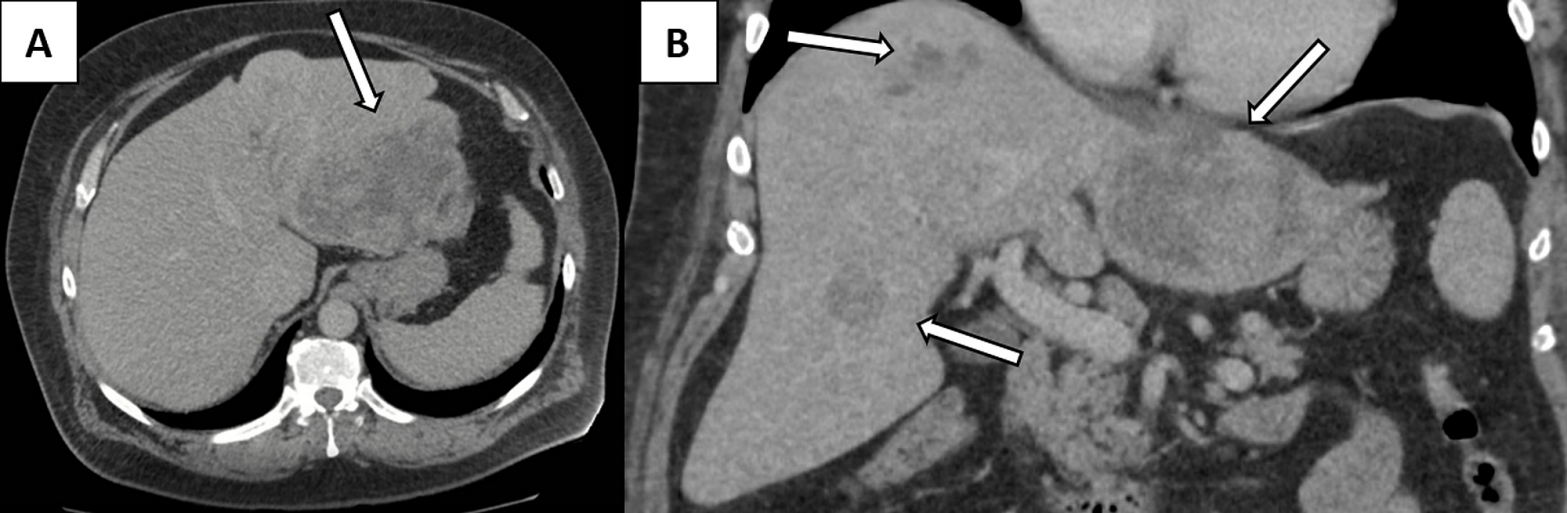
The initial CT of the patient illustrating the multiple liver lesions in the axial (A) and coronal (B) planes which were initially diagnosed at liver abscesses.

The initial bloods of the patient demonstrated that the white cell count (WCC) was 34 x 109/L and C-reactive protein was 350 (normal <3). Blood cultures and foot swabs grew *Morganella morganii* and *Proteus mirabilis*. The hepatitis screen was negative. Tumour markers were not elevated (alpha-fetoprotein 3.3 ug/L (normal <7), carcinoembryonic antigen (CEA) 7.0 ug/L, cancer antigen (CA) 19.9 6.4 (normal <35). International normalized ratio (INR) was not deranged. The abdominal ultrasound and computed tomography (CT) revealed multiple hypoechoic liver lesions suspicious for abscesses, with a differential diagnosis of malignant lesions (Figure 1). There was no imaging or biochemical evidence of cirrhosis. Echocardiogram revealed vegetations on both the mitral and aortic valves.

On presentation, this gentleman was diagnosed with liver abscesses and infective endocarditis secondary to an infected foot ulcer. He was treated with broad-spectrum antibiotics and transferred to a cardiac unit for emergent aortic and mitral valve replacement. Repeated aspiration of the liver lesions failed to grow bacteria and the tissue was not sent for histology for six months. He was treated with a prolonged course of intravenous antibiotics and returned to his rural home on oral antibiotics. Imaging at three months revealed no resolution of the lesions. Six months later, the patient presented with haematemesis. Upper endoscopy revealed a bleeding gastric ulcer that could not be controlled by endoscopic means. He was taken for urgent laparotomy at the regional hospital. The surgeon diagnosed a 10-centimetre left-sided liver lesion eroding the stomach resulting in bleeding. Necrotic material was exuding from the lesion into the stomach, so again this was diagnosed as an abscess. The liver exhibited no signs of cirrhosis, however there were five other lesions scattered through the liver. The main lesion was disconnected from the stomach and the defect in the stomach was closed. The patient was transferred ventilated to our tertiary facility for ongoing care. Imaging continued to maintain the diagnosis of liver abscess. The attending surgeon requested repeat biopsy of the left-sided liver lesion. It was highly unusual for a liver abscess to not respond to antibiotic therapy. Biopsy confirmed hepatocellular carcinoma that had ruptured into the stomach and that the patient had multifocal malignancy.

**Figure 2:**
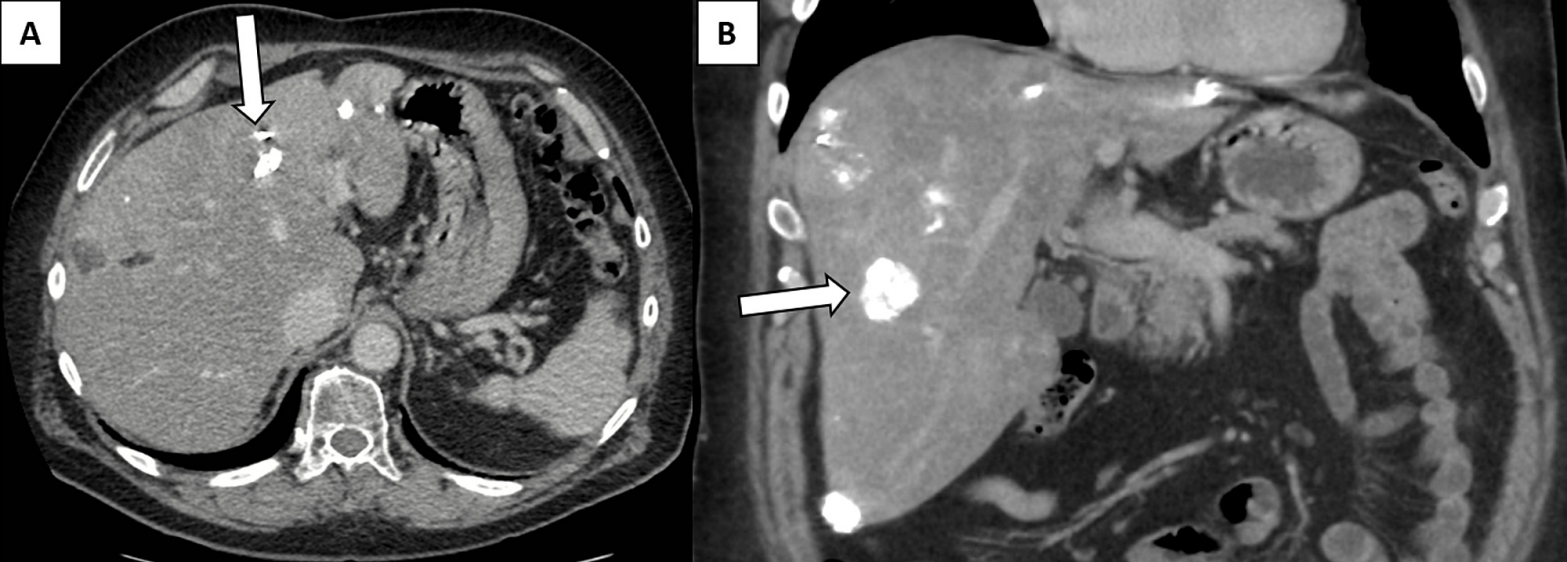
The most recent CT of patient after four years from the initial diagnosis and 11 transarterial chemoembolization procedures. The axial (A) and Coronal (B) planes demonstrate the treated multifocal hepatocellular cancer taking up Lipiodol with resolution of left sided disease.

The patient’s condition continued to improve but due to the ruptured tumour and the multifocal nature of the cancer, a plan was made for his palliation. He underwent arterial embolization of the eroding left liver lesion with gelfoam to arrest ongoing bleeding. He also received external beam radiation to the stomach and liver. He had a remarkable result and there was complete resolution of the left-sided disease. The other liver lesions were then treated with transarterial chemoembolisation (TACE). The patient has maintained a good liver function to enable him to undergo 11 TACE procedures with the latest imaging demonstrated in Figure 2.

The patient is now four years after his initial presentation and is symptom-free, enjoying an excellent quality of life. Regular imaging has revealed multiple recurrent hepatic tumours and loco-regional nodal disease. He has so far received 11 individual TACE treatments and further external beam radiation for local disease control, all of which have been well tolerated. The patient continues to be independent with activities of daily living.

## Discussion

HCC frequently has a delayed presentation with abdominal pain from rupture, erosion, and bleeding [5,6]. These advanced lesions, however, can occasionally be mistaken for liver abscesses as was the situation in this case. In this unusual case, the presence of a concurrent infective process of the foot ulcer leading to infective endocarditis distracted the clinicians from the fact that two unrelated diagnoses were present. The second confounding problem in this case was geography. This patient lived in an isolated part of the country and due to his multiple problems was treated in four different hospitals leading to a lack of continuity of care. The issue of remoteness is an ongoing issue and clinicians need to remain hypervigilant that these patients do not suffer as a result. If the initial ultrasound-guided aspirates were also sent for histology, it might have expedited the diagnosis and treatment which is an important learning point.

HCC has a poor prognosis with a median survival of 12 to 17 months [7,8]. Survival can be improved by early detection and management [9]. Therefore, it is paramount to investigate a patient with a liver lesion for HCC to allow early referral to a specialist centre. A delay in diagnosis can allow the progression of a potentially resectable lesion. Other treatment options include liver transplantation, transarterial chemoembolization, ablation, and chemotherapy such as sorafenib [10]. The literature demonstrates a significant but modest improvement in survival of patients treated with TACE [7]. However, there is a wide variety in the protocols for the application of TACE. Most patients would undergo two to three TACE procedures and 11 is certainly very uncommon. Therefore, this case demonstrates the potential of TACE to prolong life well beyond the expected prognosis.

## Conclusions

The presentation of hepatocellular carcinoma can be highly variable and can be misdiagnosed as liver abscess. A high clinical suspicion should be employed when the disease fails to resolve despite adequate treatment. Early referral to a specialist centre can improve collaboration and outcomes of patients with a liver lesion. Modern palliative HCC treatments can successfully achieve local disease control and provide the patient with an adequate quality of life.
